# Renal manifestations of recreational drugs: A narrative review of the literature

**DOI:** 10.1097/MD.0000000000031888

**Published:** 2022-12-16

**Authors:** Lakshmi Kannan

**Affiliations:** a Department of Nephrology, Pikeville Medical Center, University of Pikeville Kentucky College of Osteopathic Medicine, Pikeville, KY.

**Keywords:** acute kidney injury, glomerular disease, illicit drugs, renal injury, rhabdomyolysis

## Abstract

Drug abuse has become a major problem of the modern world where drug-induced kidney injury can be caused by both prescribed drugs for clinical conditions and illegal (illicit) drugs or drugs of abuse. Heroin, cocaine, nicotine and alcohol are the most commonly abused drugs but with the emergence of various synthetic drugs, numerous novel descriptions of their nephrotoxic effects have been described. This review summarizes the key renal manifestations of recreational drugs as reported in case reports and case. A comprehensive review of published case reports and case series in English language of renal toxicity related to recreational drugs/drugs of abuse was conducted using search engines like PubMed/Medline. Publications which reported renal injury with raised creatinine levels, clinically symptomatic patients, those with oliguria and with renal biopsies are chosen. The medical literature on recreational drugs is full of claims of renal complications including different glomerular diseases, acute kidney injury, rhabdomyolysis, interstitial nephritis, and debilitating irreversible conditions like renal infarction and end stage renal disease, even though the pathogenesis of drug- related renal manifestations are not available for all the newer agents. The outcome of this review paper will help multidisciplinary physicians to understand the renal side effects of recreational drugs, their pathophysiology, and most importantly, the clinical presentations of renal dysfunction in relation each drug. Emphasizing these adverse effects will prevent future unfavorable outcomes.

## 1. Introduction

Substance abuse is common with a lifetime prevalence of 10% in the general American population, as stated in the National Survey on Drug Use and Health conducted by the Substance Abuse and Mental Health Service Administration (SAMHSA).^[1]^ Generally, recreational drugs are divided into nine categories according to Drug Enforcement Administration: depressants, stimulants, hallucinogens, steroids, dissociative anesthetics, narcotic analgesics, inhalants, cannabis/ marijuana, and designer drugs. These drugs can have a direct or indirect effect on organ systems, of which the filtering units of the kidneys are the most affected. There is a wide range of side effects due to multiple mechanisms of injury the drugs can have- ranging from acute prerenal kidney injury (AKI) to severe functional and structural injuries. Rhabdomyolysis is the most common cause of AKI among users, due to immobilization and muscular breakdown of exertion. Vasoconstriction and ischemic renal injury are seen in cocaine and methamphetamine users.^[2]^ Glomerular injury, such as minimal change disease, focal segmental glomerulosclerosis (FSGS) is seen in heroin and cocaine abusers.^[3]^ Adulterants such as levamisole are known to cause anti-neutrophil cytoplasmic antibody (ANCA) vasculitis.^[4]^

This review discusses the nephrotoxic effects of stimulants, hallucinogens, steroids, dissociative anesthetics, narcotic analgesics, inhalants, and designer drugs, of which both the community and health care providers should become aware given their widespread use. The discussion on depressants, tobacco, e-cigarettes, alcohol, prescription opioid drugs is beyond the scope of this article.

## 2. Methods

A comprehensive search for published case reports and case series which reported renal injury in relation to the use of recreational drugs was carried out using PubMed/ Medline. Since, this is a narrative review, the only inclusion criteria were that the articles should be in English language (Table 1). The institutional review board at Pikeville Medical Center waived the need for ethical approval as this is a review of the medical literature.

### 2.1. Stimulants

Central nervous system stimulants “speed-up,” or overstimulate the body. Examples of stimulants include cocaine, crack cocaine, and amphetamines.

### 2.2. Cocaine and crack cocaine

During the second millennium, with world trade being established from China to Europe and trade routes in India, came the cocaine that kept the trade market flourishing. Cocaine (benzoyl methylecgonine) was first introduced in the United States in 1854. It was extracted from the leaves of *Erythorxylon coca*.^[86]^

#### 2.2.1. Incidence.

In the United States, the number of people using cocaine increased by 57% from 2012 to 2017. Annual deaths increased by 200%, making cocaine the leading nonopioid cause of drug overdose death according to Centers for Disease Control and Prevention.^[3]^

#### 2.2.2. Pathophysiology.

A study by Fine et al in 2007 on 193 human immunodeficiency virus patients who underwent kidney biopsy, cocaine use was present in 16 (55%) of 29 with hypertensive renal changes compared with 6 (25%) of 24 without hypertensive renal changes.^[4]^ A study by Paolo et al in a series of 40 autopsies, glomerular hyalinosis and periglomerular fibrosis was significantly higher in cocaine addicts along with a higher degree of arteriolar sclerosis, intimal and medial thickness and circumference.^[87]^ The pathophysiological basis for cocaine-related renal injury is multifactorial^[88]^-

(1) Has potent vasoconstrictive effects on vascular smooth muscle by
  a. Inhibiting catecholamine reuptake at the presynaptic nerve terminal
  b. Releasing norepinephrine and epinephrine from adrenal medulla
  c. Blocking norepinephrine reuptake in sympathetically innervated tissues
  d. Increased endothethelin-1 production resulting in decreased renal blood flow
  e. Involvement of renin-angiotensin-aldosterone system.
(2) Accelerated atherogenesis leading to renal infarction by increasing renal cellular oxidative stress and decreasing intracellular glutathione. It is also mediated by cocaine- induced stimulation of platelet aggregation and thromboxane synthesis.
(3) Accelerated and malignant hypertension which hastens the progression of chronic kidney disease to end stage renal disease.
(4) Formation of active metabolite cocaethylene which is highly toxic than cocaine alone leading to rhabdomyolysis. It could also be related to non- traumatic injury leading to acute skeletal myofibrillar degeneration and vasoconstriction leading to muscle ischemia and necrosis, or traumatic due to seizure or hyperthermia.

#### 2.2.3. Clinical characteristics.

There are several case reports suggesting cocaine- induced rhabdomyolysis (Table 1). It has been associated with accelerated and malignant hypertension as well as hastening the progression of hypertensive nephrosclerosis to end stage renal disease.^[48,89]^ Acute aortic thrombosis, renal artery thrombosis and dissection have been reported.^[5,10]^ Other clinical charactertistics include acute interstitial nephritis, thrombotic microangiopathy and chronic kidney disease.

**Table 1:** Case reports and case series on renal effects of recreational drugs.*

	Case report/case series	Age (yr)	Sex	Route of administration	Renal manifestation	Kidney biopsy findings
Cocaine with and without levamisole	Mochizuki et al, 2003.^[5]^ Fabbian et al, 2012.^[6]^ Fogo et al, 1992.^[7]^ Madhrira et al, 2008.^[8]^ Bemanian et al, 2005.^[9]^ Edmondson et al, 2004.^[10]^ Herzlich et al, 1988.^[11]^ Neynaber et al, 2008.^[12]^ McGrath et al, 2011.^[13]^ Veronese et al, 2016.^[14]^ Carrara et al, 2016.^[15]^ Garg et al, 2015.^[16]^ Veer et al, 2015.^[17]^ Gu et al, 2007.^[18]^ Mousa et al, 2016.^[19]^ Doctora et al, 2003.^[20]^ Roth et al, 1988.^[21]^	41 25 48 47 40 27 22 30 patients, 41–52 yr 49 42 34 40 39 48 and 39 41 38 39 patients	Male Male Male Male Male Male Male Males and Females Male Female Female Male Female Females Male Males and Females Males and females	Intranasal Intranasal Intranasal Smoking Smoking Smoking Smoking Oral Oral Oral Oral Smoking Smoking Oral Oral Oral Crack	AKI, hypertension AKI, hematuria AKI AKI AKI, hematuria AKI and rhabdomyolysis AKI Cutaneous vasculitis, AKI. AKI, hematuria AKI, hematuria, skin lesions AKI Ulcerative skin lesions, AKI. AKI, accelerated hypertension Rhabdomyolysis and AKI Rhabdomyolysis Rhabdomyolysis, AKI AKI	Severe arteriosclerosis/renal infarction. NA NA NA NA NA NA Broad area of complete coagulative-type necrosis with loss of all cellular elements and interstitial hemorrhage. Proximal tubules with luminal ectasia, cytoplasmic simplification, extensive loss of brush border, and many apoptotic figures. Renal artery dissection and thrombosis. NA NA NA Focal and segmental pauci-immune crescentic glomerulonephritis. Small arteries with onion-skinning and myxoid intimal change with segmental fibrinoid necrosis of vascular walls. Glomeruli fibrinoid necrosis of capillary tufts. NA NA NA NA
Amphetamines	Terada et al, 1988^[22]^ Fahal et al, 1992.^[23]^ Ginsberg et al, 1970^[24]^ Kendrick et al, 1977^[25]^ Foley et al, 1984^[26]^ Bingham et al, 1998.^[27]^ Eldehni et al, 2010.^[28]^ Cherney et al, 2002.^[29]^ Traub et al, 2002.^[30]^ Hartung et al, 2002.^[31]^ Bryden et al, 1995^[9]^ Kwon et al, 2003.^[32]^ Vakde et al, 2014.^[12]^	36 23 21 5 patients in their teens 32 30 22 20 19 17 patients aged 15-26 yr 19 18 29	Male Male Male Males Male Male Male Female Female Males and females Male Female Male	Intravenous Intravenous Intravenous Intravenous Oral Oral Oral Oral Oral Oral Oral Oral Oral	Rhabdomyolysis and AKI. AKI, rhabdomyolysis, DIC AKI AKI and Rhabdomyolysis AKI AKI AKI, hematuria, proteinuria. Hyponatremia Hyponatremia Hyponatremia Urinary retention. Hyponatremia, polyuria, renal glycosuria. AKI from molly.	Tubular degeneration and tubular obstruction with myoglobin casts. Acute tubular necrosis. NA NA Acute interstitial nephritis. NA Arterioles with fibrinoid necrosis with in sudation of fibrin and red cells to thickening of the edematous intimal with occlusion of the lumen consistent with necrotizing vasculopathy. Glomeruli- lobulation with mesangial thickening, some with focal/ segmental necrotizing lesions/ nonepithelial cells. A small vein at the corticomedullary junction was occluded by thrombus. NA NA NA NA NA NA
LSD	Mercieca et al, 1984.	2 patients, 19 and 25.	Males	Oral	AKI and rhabdomyolysis.	NA
Magic mushrooms	Austin et al, 2019.^[33]^ Beaumier et al 2019.^[13]^ Kirchmair et al, 2012.^[34]^ Raff et al, 1992.^[35]^ Bickel et al, 2005.^[36]^	15 61 2 patients, aged 51 and 47 yr 20 25	Male Female Male and Female Female Male	Oral Oral Oral Oral Oral	AKI AKI AKI AKI AKI, rhabdomyolysis.	Severe interstitial fibrosis, flattened tubular epithelial cells, dilated tubular lumen and loss of brush border. NA Acute interstitial nephritis and tubular necrosis. Ischemic tubular necrosis. NA
PCP	Patel et al, 1980^[37,38]^ Akmal et al, 1981^[39]^ Barton et al, 1980.^[40]^	8 patients. 25 out of 1000 patients 2 patients	Males Males and Females Males	Oral Oral Oral	AKI and rhabdomyolysis. AKI, rhabdomyolysis AKI, rhabdomyolysis.	NA NA NA
Ketamine	Selby et al, 2008.^[41]^ Chen et al, 2011.^[42]^ Tran et al, 2014.^[43]^ Shahani et al, 2007.^[44]^ Lai et al, 2012.^[43]^ Wu et al, 2012.^[45]^ Lee et al, 2015.^[46]^ Chu et al, 2007.^[47]^	26 4 patients, 20–24 yr old 24 9 patients 6 patients 4 patients 9 patients, aged 28-42 yr 10 patients aged 20–30 yr.	Male Males and females Male Males Males and females Males and females Males and females Male and female.	Intranasal Intranasal Intravenous Intravenous Intravenous Intravenous Intravenous Intravenous	AKI, hematuria Anuria, hematuria(cystitis), bilateral hydronephrosis. Cystitis and B/L hydronephrosis. Dysuria, increased frequency, and urgency. Urinary retention, bilateral hydronephrosis. Dysuria, increased frequency. Increased urinary frequency, hematuria. Dysuria	Tubular injury NA NA NA Bladder biopsy- ulcerative cystitis. Chronic cystitis. Acute cystitis with eosinophilic infiltration. NA
Anabolic androgenic steroids	Herlitz L et al, 2010.^[48]^ Harrington et al, 2011.^[45]^ Almukhtar et al, 2015.^[49]^ Sandhu et al, 2002.^[50]^ Habscheid et al, 2007.^[51]^ Daher et al, 2009.^[52]^ Luciano et al, 2014.^[53]^ Alkhunaizi et al, 2016.^[54]^	10 patients, age ranging from 28 to 49 yr 38 4 patients, ages 21–26 yr 3 patients, aged 27, 27 and 28. 28 yr 2 patients, 21 and 30 yr old 28 19	Males Male Males Males Male Males Male Male	Injectable testosterone, oral methyl-1-testosterone Oral Intravenous Oral Oral Oral Oral Oral	AKI, nephrotic syndrome. Nephrotic syndrome AKI AKI, Rhabdomyolysis. AKI AKI AKI, obstructive jaundice AKI	FSGS- 4 had perihilar lesions and 3 had collapsing lesions. FSGS Flattened tubular epithelium with loss of nuclei and epithelial desquamation and blebbing. NA NA Acute tubular necrosis and inflammatory interstitial nephritis. NA Bile casts within the tubular lumen, filamentous bile inclusions within the tubular cells.
Heroin	Kilcoyne et al, 1972.^[37]^ Rao et al, 1974.^[55]^ Llach et al, 1979.^[16]^ Uzan et al, 1988.^[40]^ Sahni et al, 2005.^[56]^ Cunningham et al, 1980.^[14]^ Faria et al, 2003.^[57]^ Turgutalp et al, 2012.^[58]^ Manner et al, 2009.^[34]^ Tan et al, 1995.^[59]^ Cooper et al, 2013.^[18]^ Bautista et al, 2015.^[60]^	8 patients. 14 patients 19 patients, 20-47 yr 13 patients. 23 patients, 18-45 yr old 19 patients 29 9 patients 32 37 42 56	Males and Females Males and Females Males and Females Males and females Males Males and females Male Males and females Female Female Male Male	Oral Oral Intravenous Intravenous and sniffing Intravenous Intravenous Intravenous Intravenous Skin popping Skin popping Intravenous Intravenous	Nephrotic range proteinuria. Nephrotic range proteinuria Nephrotic range proteinuria AKI, rhabdomyolysis, nephrotic range proteinuria AKI and nephrotic range proteinuria AKI, nephrotic syndrome Nephrotic syndrome CKD CKD AKI with sub-nephrotic range proteinuria. AKI, severe metabolic alkalosis. Urine with crystals that resembled broomsticks. AKI	Focal membranoproliferative glomerulonephritis and binding of IgM and B1C by electron and immunofluorescence microscopy. FSGS with focal glomerular deposition of IgM and β_1_C/β_1_A globulin. Membranous glomerulonephritis Acute tubular necrosis. Glomerulonephritis without glomerulosclerosis. NA Sclerosing glomerulonephritis Membranoproliferative glomerulonephritis with IgM and C3 deposition. Minimal change disease with IgA deposition. AA amyloidosis NA Diffuse acute tubular injury with intratubular crystals- finely granular to fluffy deeply basophilic appearance. NA
Narcotics (non-heroin) T’s and Blues Syndrome OPANA-ER	May et al, 1986.^[61]^ Hunt et al, 2017.^[21]^ Jabr et al, 2016.^[62]^ Miller et al, 2014.^[63]^	3 patients. 3 patients, 24,28 and 48. 37 18 patients, 23-52 yr	Males and females Males and 1 female Male Males and females	Intravenous Intravenous Intravenous Intravenous	Nephrotic syndrome AKI, anemia, thrombocytopenia, peripheral smear showing schistocytes. AKI, hemolytic anemia. AKI, hemolytic anemia	Focal to diffuse segmental or global glomerulosclerosis. Electron microscopy- glomerular visceral epithelial cell foot process effacement and microvillus formation. TMA with focal endothelial swelling involving larger arteries, extensive acute tubular injury. NA NA
Toluene (Glue sniffing)	Taverner et al, 1988^[64]^ O’Brien et al, 1971.^[44]^ Taher et al, 1974.^[43]^ Moss et al, 1980.^[65]^ Patel et al, 2008.^[66]^ Lemarroy et al, 2012.^[67]^	19 23 27 32 38 22 patients, mean age-23 yr	Male Male Male Female Female Males and females	Inhalation Inhalation Inhalation Inhalation Inhalation Inhalation	AKI AKI AKI, hypokalemia with hyperchloremic metabolic acidosis. Severe hyperchloremic metabolic acidosis Hypokalemia, distal renal tubular acidosis. Oliguria Severe metabolic acidosis.	Severe tubule-interstitial nephritis. NA NA NA NA NA
Bath salts	Regunath et al, 2012^[68,69]^ McNeely et al, 2012.^[70]^ Sutamatewagul et al, 2014.^[71]^ Murray et al, 2012. Rhidian et alm 2013.^[72]^ Adebamiro et al, 2012.^[73]^ Borek et al, 2012.^[74]^ ^[70]^	NA 29 37 40 25 35 25	Male Male Male Male Male Male Male	Oral Snorting Intravenous Snorting Oral Oral Intravenous	AKI AKI, hyperuricemia, rhabdomyolysis AKI, rhabdomyolysis. Hyperthermia, rhabdomyolysis, acidosis. Oligoanuric AKI. Rhabdomyolysis, hyperuricemia, and metabolic acidosis. Hyperthermia, AKI requiring dialysis.	NA NA NA NA NA NA NA
Synthetic cannabinoids	Ergul DF et al	6 patients, ages from 21 to 28 yr.	5- males	Bonzai (1-naphthalenyl of methanol).	AKI, rhabdomyolysis.	NA.
	Srisung et al	3 patients 31, 32 and 33 yr old.	1-female.	Oral	AKI, rhabdomyolysis.	Acute tubular injury, tubular cell apical blebbing, and cytoplasmic vacuolization.
	Kazory et al	22	Males	Smoking	AKI	Acute tubular necrosis with focal tubular atrophy and flattened epithelium.
	Curtis et al	21	Male	Smoking	AKI	NA
	Zhao et al	39	Male	Smoking	AKI	NA
	Bhanushali et al	4 patients, age ranging from 20 to 30 yr	Male	Smoking	AKI	Acute tubular injury, tubular dilation, and tubular epithelial cell vacuolization. Few crystal depositions of calcium oxalate.
	Kamel et al	65	Males	Bonzai	AKI	NA
	Sinangil et al	42	Male	Smoking	AKI, uveitis	Diffuse inflammatory infiltrates with lymphocytic predominance with tubulitis (TINU).
	Buser et al	15- 27 yr	Male	Oral	AKI.	NA
	Gudsoorkar et al	26	Males	Smoking	Rhabdomyolysis, AKI.	NA
	Sherpa et al	45	Male	Smoking	AKI, hypernatremia, hypokalemia, severe hypophosphatemia.	NA
	Thornton et al	26	Male	Smoking	AKI	Rare globally sclerotic glomeruli.

AKI = acute kidney injury, FSGS = focal segmental glomerulosclerosis, NA = not available, TMA = thrombotic microangiopathy.

* Only case reports and case series in English are included.

Cocaine abuse in pregnancy can cause a decrease in fetal arterial flow, urine output, bladder cycle, higher resistance index of renal artery, thickening of the interlobular arterial wall of the fetal kidney and luminal narrowing.^[90,91]^

### 2.3. Cocaine with levamisole

Cocaine, in recent days, has been mixed with adulterants such as levamisole, dexamisole, and fentanyl. Levamisole, which is a discontinued anti-helminthic is the most common adulterant and when mixed with cocaine potentiates its effects.

#### 2.3.1. Incidence.

69% of cocaine is adulterated with levamisole as reported by Drug Enforcement Administration. In an analysis of cocaine users in Seattle, Washington, approximately 80% of users who tested positive for cocaine also tested positive for levamisole.^[92]^

#### 2.3.2. . Pathophysiology.

A case series of 30 patients with ANCA-associated vasculitis in relation with cocaine and levamisole has been reported (Table 1). The pathophysiology of levamisole-induced vasculitis involves^[93]^:

1. The action of catecholamines on neural synapses enhances the reuptake inhibition effect of cocaine.
2. Immunomodulation by promoting neutrophil mobility and chemotaxis, enhancing dendritic cell maturation, and promoting T cell proliferation that induces autoimmunity and vasculitis.

#### 2.3.3. Clinical characteristics.

Cocaine with levamisole has immunomodulatory properties and causes crescentic pauci-immune GN, positive for both MPO and PR3 antibodies. Additionally, many patients also had antinuclear antibody positivity and low complements. A case report has shown an association of anti-glomerular basement membrane disease.^[94]^

### 2.4. Amphetamines

Amphetamines were first synthesized in 1887 and were used in the medical community to raise blood pressure and stimulate the central nervous system. During World War II, it was widely distributed among soldiers to combat fatigue and improve mood and endurance.^[94]^

#### 2.4.1. Incidence.

Among the illicit drugs, amphetamines remain a popular recreational drug in the world. According to SAMHSA, more than 150000 emergency department visits were decscribed from toxicity from amphetamines and its analogues.^[1]^

#### 2.4.2. Pathophysiology.

The renal complications as a result of amphetamine use are associated with hyperthermia and fibrinolysis. Microvascular obstruction secondary to disseminated intravascular coagulation, myoglobinuria and systemic hypotension lead to AKI.^[95]^

#### 2.4.3. Clinical characteristics.

There are several case reports of rhabdomyolysis, disseminated intravascular coagulation, and AKI in the literature as shown in Table 1.

### 2.5. Hallucinogens

Hallucinogens are a group of drugs that alter a person’s awareness of their surroundings as well as their own thoughts. Drugs like LSD, psilocybin, and MDMA (ecstasy) are classic hallucinogens.

In ancient Greece, a special form of mead (fermented honey) was used to induce vision known as mysteries. With this, came the cultivation of plants (peyote cactus, fly agaric and cannabis) containing entheogens (natural chemicals that induce hallucinations) throughout the world.^[96]^

The percentage of people who were hallucinogen users in the past year increased from 0.8 percent (or 1.7 million people) in 2015 to 1.5 percent (or 3.1 million people) in 2019.^[1]^

### 2.6. LSD

LSD (D-lysergic acid diethylamide) was first accidentally discovered in the labs by Dr Albert Hofmann in 1943. Eventually, U.S. military and CIA used it as a possible “truth drug” to induce prisoners to talk. Non-therapeutic use of LSD occurred in the late 1950s and 1960s.

#### 2.6.1. Incidence.

In a survey conducted in 2010 by National Survey on Drug Use and Health, 23 million US residents reported using LSD at least once.^[97]^

#### 2.6.2. Pathophysiology.

There are not a lot of research or studies on LSD but it has been noted that LSD-induced hyperthermia leads to rhabdomyolysis based on a case report by Berrens et al in 2010.^[98]^

#### 2.6.3. Clinical characteristics.

Patients can present with hypertension, tachycardia, agitation, seizures and AKI. There are also case reports of metabolic acidosis and rhabdomyolysis.

### 2.7. Magic mushrooms

Out of the 100,000 or more species of mushrooms worldwide, more than 100 are toxic. Identifying the specific species of mushrooms is important as there is a specific treatment for some mushrooms. Magic mushrooms (*Psilocybe semilanceata*) have become a popular form of substance abuse among young people. This fungus contains a hallucinogenic agent psilocybin, which resembles LSD.^[33]^ Other mushroom intoxications include amatoxin syndrome and orellanine syndrome due to *Cortinarius*.

#### 2.7.1. Incidence.

Magic mushroom is very popular in rave parties and college parties. Hallock et al based on a survey of 409 college students reported that 29.5% of responded experimented with psilocybin- containing hallucinogenic magic mushrooms.^[33]^ Another study indicated that an estimated 21 million people in the US used magic mushrooms in the past.^[68]^

#### 2.7.2. Pathophysiology.

##### 2.7.2.1. Pathophysiology involves.

1. Once ingested, psilocybin is dephosphorylated by alkaline phosphatase to the active metabolite psilocin.
2. Both psilocybin and psilocin have affinity for serotonergic receptors, which are responsible for hallucinatory properties.
3. Mushrooms of the *Cortinarius* genus contain the toxin orellanine that causes inhibition of protein, RNA, and DNA synthesis.
4. It produces an orthosemiquinone radical that can lead to oxidative stress and direct toxicity if the renal tubular epithelium causes tubular necrosis, interstitial nephritis, and fibrosis.

#### 2.7.3. Clinical characteristics.

Ingestion of magic mushrooms is regarded as having a low potential for arm. The most commonly reported adverse effects are anxiety, agitation, confusion, impaired concentration and judgment. Renal manifestations include renal failure from severe dehydration to nephritis and multiorgan failure.

### 2.8. MDMA

The first use of methamphetamine (injectable form of amphetamine) began in the 1960s. It is a drug which by increasing dopamine in the central nervous system, and causes dependence. Other street names are Speed, ice and crank.

#### 2.8.1. Incidence.

Currently, methamphetamines are commonly used by students and unemployed people in their 20s and 30s. The percentage who was past year methamphetamine users increased from 0.5 percent (or 1.1 million people) in 2016 to 0.8 percent (or 1.7 million people) in 2019.^[1]^

#### 2.8.2. Pathophysiology.

Methamphetamine, especially MDMA, causes AKI through several mechanisms^[99]^:

1. Myoglobinuria- associated tubular injury secondary to rhabdomyolysis.
2. Hyperuricemia
3. Malignant hypertension
4. Serotonin syndrome
5. Isolated proximal tubular injury
6. Hyponatremia is caused by HMMA (4-hydroxy 3- methoxymethamphetamine)- a metabolite of MDMA; can be dilutional in nature due to excessive water or other hypotonic beverage intake (psychogenic polydipsia); can be due to inappropriate secretion of arginine vasopressin.

A pure form of 3,4-methylenedioxymethamphetamine, called “Molly” acts by enhancing the release of serotonin, dopamine and norepinephrine. Though it is a popular club drug as it produces euphoria, it is associated with serious side effects including rapid multiorgan failure, hyponatremia and rhabdomyolysis (Table 1).

#### 2.8.3. Clinical characteristics.

Renal manifestations of MDMA (3.4-methylenedioxymethylamphetamine) (ecstasy) include rhabdomyolysis, malignant hypertension, hyponatremia, necrotizing vasculitis, thrombotic thrombocytopenic purpura and rapidly progressive glomerulonephritis. Gupta et al in 2018 published the first case report of crystal meth induced acute renal cortical necrosis,^[86]^ in addition, to a myriad of cardiovascular and cerebrovascular complications like malignant hypertension, arrhythmias, aortic dissection, myocardial infarction, stroke, and cardiomyopathy.^[93]^

### 2.9. Dissociative anesthetics/ hallucinogens

Dissociative anesthetics include drugs like phencyclidine (PCP) (also belong to dissociative hallucinogen group), ketamine, and dextromethorphan that inhibit pain by dissociating the brain’s perception of the pain.

### 2.10. PCP

PCP was first developed in the 1950s as a general anesthetic for surgeries. It was soon abandoned due to unwanted side effects, including psychosis and dysphoria. Due to its dissociative effects, it gained popularity as a drug of abuse.^[100]^ Various case reports of PCP and rhabdomyolysis/acute renal failure since the 1970s are available in the literature (Table 1).

#### 2.10.1. Incidence.

Common street names for PCP are the peace pill, angel dust, crystal joints, rocket fuel, sawgrass, zoom, the sheets, and elephant tranquilizer. There were 75,538 emergency department visits in 2011 due to PCP, according to the Drug Abuse Warning Network. This was up 400% from 2005 (14,825) based on Drug Abuse Warning Report.

#### 2.10.2. Pathophysiology.

PCP is a noncompetitive antagonist to the NMDA receptor, which causes analgesia, anesthesia, cognitive defects, and psychosis. PCP blocks the uptake of dopamine and norepinephrine, leading to sympathomimetic effects such as hypertension, tachycardia, bronchodilation, and agitation.

#### 2.10.3. Clinical characteristics.

Rhabdomyolysis, hypoglycemia, seizures, hypertensive crisis, coma, and trauma are several of the complications that can arise with PCP use.

### 2.11. Ketamine

**Figure 1.**
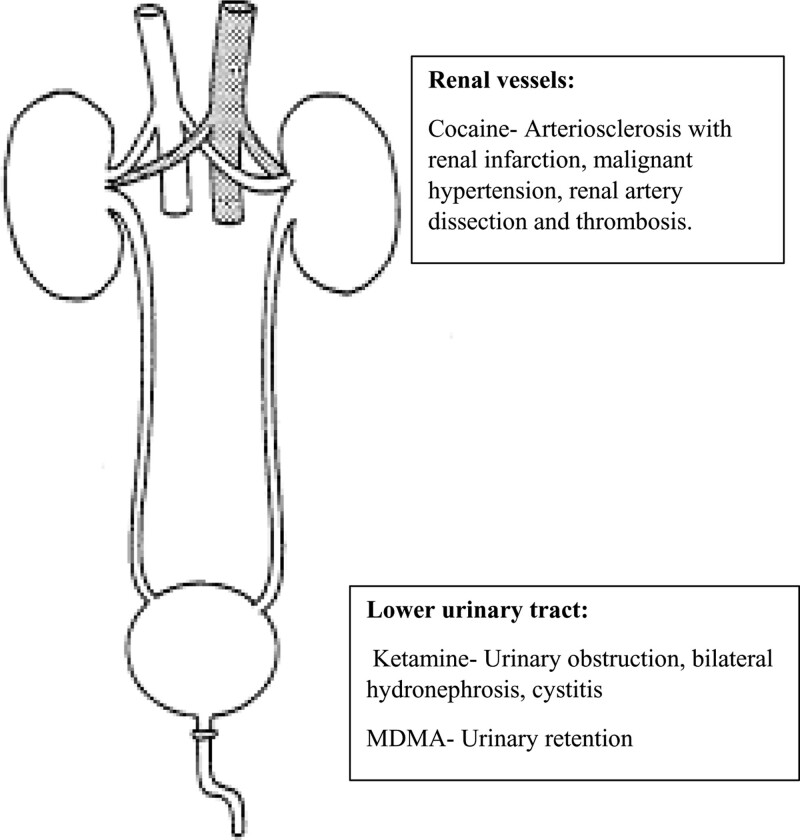
Site of action of recreational drugs in the urinary system.

Ketamine is an anesthetic agent (N-methyl-D-aspartic acid receptor antagonist) that is widely used in veterinary medicine and was first synthesized in 1962. As a drug, it causes mood elevation, visual hallucinations, and derealization. Ketamine use has been mainly associated with urinary tract abnormalities, such as inflammatory cystitis, hematuria, and an obstructive picture with hydronephrosis (Fig. 1 and Table 1).^[101]^

#### 2.11.1. Incidence.

The highest prevalence of recreational ketamine use—0.9%) was reported at the end of 2019.^[76]^

#### 2.11.2. Pathophysiology.

Most of the ketamine metabolites are excreted in to urine by the kidney, only 1% to 5% is eliminated via fecal excretion. Pathophysiology of urinary tract dysfunction involves^[41]^

1. Formation of gelatinous material from ketamine metabolites (norketamine in the hepatic microsomal system to hydroxynorketamine) that precipitates in the pelvicalyceal systems and causes obstructive renal failure.
2. Bladder epithelial dysfunction with urinary leak, mast cell activation and neurogenic inflammation.
3. IgE-mediated inflammation and hypersensitivity.

#### 2.11.3. Clinical characteristics.

Case reports have shown development of inflammatory cystitis with low volume bladders (Table1), bilateral hydronephrosis and AKI but kidney biopsy had no intrinsic renal pathology.

## 3. Steroids

### 3.1. Anabolic-androgenic steroids

Anabolic androgenic steroids (AAS) include testosterone and its synthetic derivatives, which have been used illicitly by athletes and individuals who wish to increase muscle mass. These substances have been in use since the 1950s.^[3]^

#### 3.1.1. Incidence.

The lifetime prevalence of AAS use worldwide is estimated to be 1% to 5%.^[102]^ The prevalence in males is 6.4% compared to 1.6% in females.^[89]^

#### 3.1.2. Pathophysiology.

The pathophysiology of renal injury is not yet well established,^[5]^ but

1. hyperfiltration injury is an important factor in patients with markedly elevated lean body mass.
2. Direct toxic effect of AAS on podocytes.
3. Stimulation of renin-angiotensin-aldosterone system
4. Enhancing endothelin, reactive oxygen species, and inflammatory cytokines (TNF-α, IL-1b and IL-6) production is postulated.

#### 3.1.3. Clinical characteristics.

Nephrotoxicity of AAS is only presented in the case reports presented in Table 1. Kidney biopsy from 10 long-term AAS abusers who presented with elevated creatinine and proteinuria showed FSGS (4 out of 10 had perihilar lesions of FSGS, 3 had collapsing lesions) with 69% podocyte foot process effacement.^[101]^

### 3.2. Narcotic analgesics

Narcotic analgesics like heroin and non- heroin substances relieve pain, induce euphoria, and create mood changes.

### 3.3. Heroin, non-heroin substances

Indians, Assyrians, and Egyptians began to cultivate opium from opium poppy. It was used only by the upper classes to relax and pass time. The isolation of morphine from opium started in 1804 as a way to alleviate the pain of war veterans at the time of the Civil War.^[96]^ Later, cocaine, heroin and other opiates were synthesized and marketed as nonaddictive alternatives to morphine

### 3.4. Heroin

Heroin is the most widely abused opiate in the United States (US). It is extracted from morphine, which can be injected, inhaled, or smoked. The purity of heroin depends on the presence of other drugs or substances called adulterants (commonly used are sucrose, dextrose, mannitol, lactose, starches, powdered milk, caffeine, lidocaine, procaine, methapyrilene, and strychnine).^[76]^

#### 3.4.1. Incidence.

The first known case of nephropathy, term known as heroin-associated nephropathy (HAN), was seen in the early 1970s in New York.^[57]^

#### 3.4.2. Pathophysiology.

Heroin abuse can lead to AKI^[103]^

1. due to dehydration, exhaustion, rhabdomyolysis, and urinary retention.
2. Intravenous administration can result in the spread of hepatitis B and C and human immunodeficiency virus which can eventually lead to GN.
3. Skin spit can cause amyloidosis as reported in a case by Cooper et al who found serum amyloid A protein depositis in a heroin abuser of 18 years.^[104]^
4. Chronic heroin use can lead to focal glomerulosclerosis with glomerular IgM deposition resulting in nephrotic syndrome.

#### 3.4.3. Clinical characteristics.

Renal complications range from acute kidney injury from rhabdomyolysis which carried a good prognosis to acute glomerulonephritis, and FSGS in heroin- associated nephropathy (HAN) (Table 1) whereas Turgutalp et al reported a patient with minimal change disease with IgA-C3C4 + and IgG1 + deposition.^[58]^ Do Samerio et al reported MPGN type I to be most dominant disease in Caucasian heroin abusers.^[57]^ Recently endocarditis associated glomerulonephritis has been increasingly seen in IV heroin users. It is almost always right- sided and is associated with crescents in 50% of the cases, 25%- 30% with ANCA.

### 3.5. Non-heroin abuse

*T’s and Blues syndrome*- The tablet form of pentazocine is combined with the antihistamine, tripelennamine, dissolved in water, and injected intravenously. This combination is called “T” and “Blue.”^[68]^

**Figure 2.**
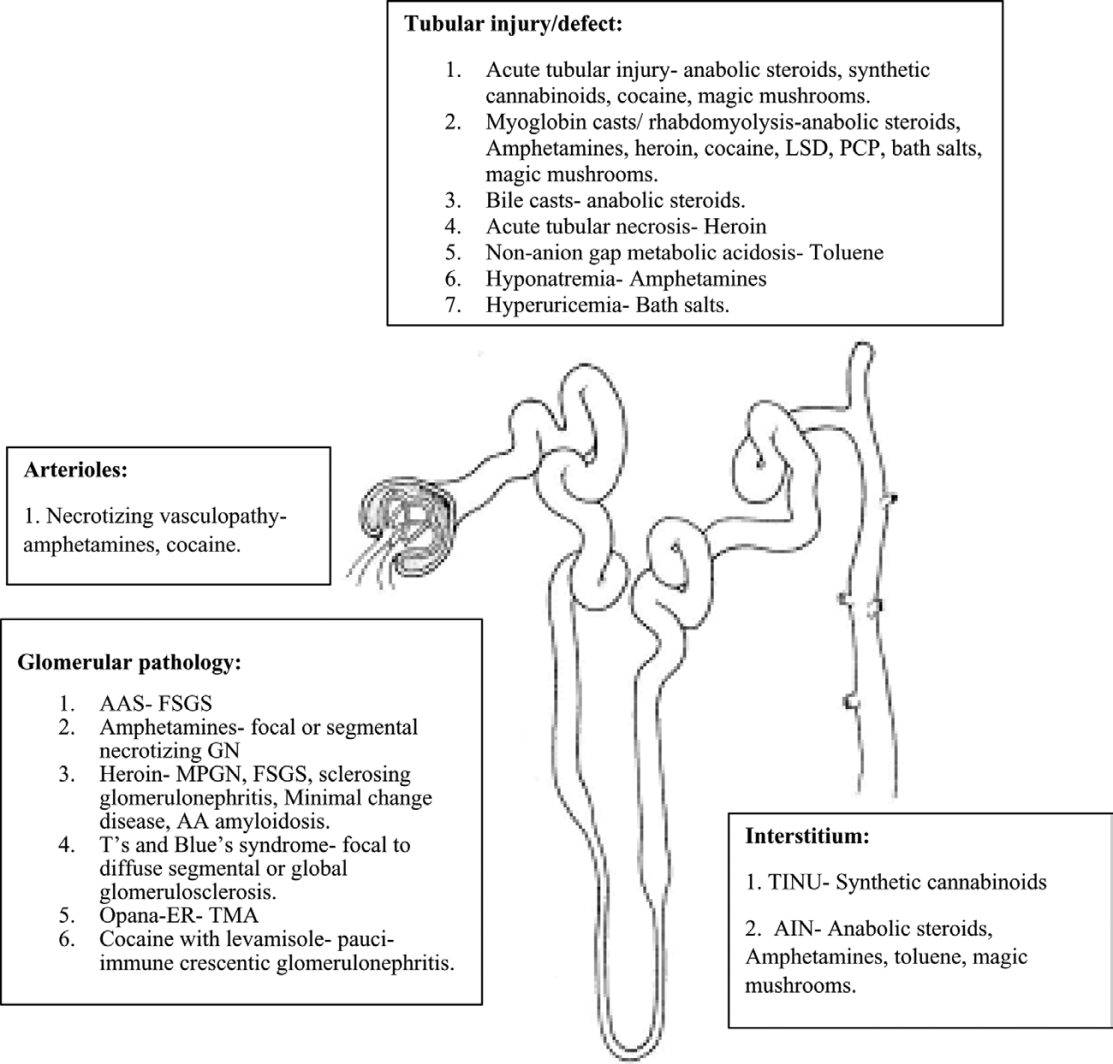
Site of action of recreational drugs in the nephron.

*Oxymorphone/ OPANA ER*- In recent years, there has been an increase in the misuse of adulterated oxymorphone injected by mixing the pill with water. This drug has been associated with thrombotic microangiopathy. A case series by Miller et al reported that 9 of 18 patients who used OPANA-ER have developed thrombotic thrombocytopenic purpura and AKI (Table 1 and Figure 2).^[75]^

### 3.6. Inhalants

Inhalants provide mind-altering effects. Examples include toluene, paint thinners and various anesthetic gases.

### 3.7. Toluene (Gas sniffing)

Toluene is an aromatic hydrocarbon that has been used in industry as an organic solvent. The “sniffing” of toluene in paint, paint thinners, and glue has been primarily associated with neurological complications, but since the early 1970s, several case reports of severe renal complications have been described, as shown in Table 1.

#### 3.7.1. Incidence.

As a recreational drug, toluene is most often inhaled nasally (glue sniffing, huffing) and produces acute neurological effects such as euphoria.

#### 3.7.2. Pathophysiology.

The hallmark of toluene intoxication is type I RTA and muscular weakness duet o hypokalemia (hypokalemic paralysis). Pathophysiology primarily involves

1. inhibition of the generation of a hydrogen-ion gradient in the distal tubules resulting in an inability to excrete hydrogen ions as ammonium leading to type I RTA or distal RTA.
2. Overporduction of hippuric acid by toluene metabolism leading to distal RTA.
3. Increased hydrogen ion back diffusion with increased potassium movement into the tubular lumen leading to potassium wasting.
4. Hypokalemic paralysis due to an increased ratio between intra- and extracellular potassium concentrations, which alters membrane polarization and function of excitable tissues such as muscle.
5. AKI caused by vomiting, dehydration, tubular injury and rhabdomyolysis.
6. Rhabdomyolysis occurs by either direct toluene muscular injury, prolonged immobility, hypokalemia or hypophosphatemia.

#### 3.7.3. Clinical characteristics.

The initial effects of toluene inhalation are euphoria, excitement, and exhilaration. As high concentrations are reached, visual and auditory hallucinations, confusion, nausea, vomiting, and loss of self-control are seen. In terms of kidney involvement, microscopic hematuria and proteinuria have been found based on the case reports (Fanconi syndrome) in Table 1. Some cases of AKI, severe normal anion gap metabolic acidosis (type I RTA), hypokalemia, and muscle paralysis has also been found.^[105]^

### 3.8. Designer drugs

Bath salts, K2/Spice/synthetic cannabinoids are designer drugs, which are collectively called synthetic legal intoxicating drugs (SLIDs). They gained popularity as they are relatively cheap, are not detected on standard urine drug screens, and can produce a powerful high.

### 3.9. Bath salts

Bath salts are beta- ketone amphetamine analogs and are derived from cathinone from the “Khat” plant (*Catha edulis*).^[104]^ These drugs are sold as white- or tan- colored crystalline powders and contain mephedrone and 3,4- methylenedioxypyrovalerone (MDPV).^[106]^

#### 3.9.1. Incidence.

In 2011, “bath salt” use was related to over 20,000 emergency department visits in the US based on a report by SAMHSA.^[1]^

#### 3.9.2. Pathophysiology.

Pathophysiology of renal toxicity of bath salts involves^[69]^

1. Inhibition of monoamine reuptake (dopamine, norepinephrine) that results in hyperthermia and rhabdomyolysis.
2. Serotonin syndrome.
3. Direct drug effects
4. Norepinephrine and dopamine- induced vasoconstriction (renal ischemia)

#### 3.9.3. Clinical characteristics.

Bath salt preparations are pharmacologically similar to amphetamines, which can cause sympathomimetic features like anxiety, agitation, and palpitations. The renal manifestations of bath salts include mild rhabdomyolysis and hyperuricemia. In severe cases, multiorgan failure with anuric AKI that requires continuous renal replacement therapy has been known to occur K2/.

### 3.10. Spice/synthetic cannabinoids

Synthetic cannabinoids have cannabis- or marijuana- like effect. It was initially produced in the laboratories for research purposes but has become popular as “Spice” or “K2.” The main categories of synthetic cannabinoids include classic cannabinoids (with a dibenzopyran ring), nonclassic cannabinoids (cycloheylphenols), naphthylmethylindoles, naphthopyrroles, naphthylmethylindenes, naphthylindoles, phenylacetylindoles, methanandamine and other synthetic analogues of endogenous eicosanoids.^[10]^

#### 3.10.1. Incidence.

Among people aged 12 or older, the percentage who were past year marijuana users increased from 11.0 percent (or 25.8 million people) in 2002 to 17.5 percent (or 48.2 million people) in 2019.^[1]^

#### 3.10.2. Pathophysiology.

The exact mechanism of nephrotoxicity of synthetic cannabinoids is not exactly known, but several reports have documented^[78]^

1. Calcium oxalate crystal deposition in kidney biopsy samples as synthetic cannabinoids is ingested with plants which may have oxalogenic potential.
2. Cannabinoid hyperemesis syndrome resulting in extreme hypovolemia and dehydration leading to AKI.^[107]^
3. Derangements in the kidney endocannabinoid system (low-level expression of the kidneys (low level expression of CB1 and CB2 receptors in renal podocytes, endothelial cells, mesangial cells and proximal tubules).^[108]^
4. Potential adulteration with noncannabinoid nephrotoxic contaminants.

#### 3.10.3. Clinical characteristics.

The first report of acute kidney injury (AKI) in patients with use of synthetic cannabinoids was in 2012 in Wyoming. Further investigation led to identification of 16 additional cases of AKI in six different states. Toxicological analysis identified a fluorinated synthetic cannabinoid. Kidney biopsy in these patients revealed acute interstitial nephritis, and acute tubular injury.^[109]^

## 4. Conclusion

Based on this review, there is still a paucity of data in the literature due to under-reporting of cases, inherent problems in finding causal association between a particular drug and the development of a single renal disease, as in most cases, they do not exist as a specific or separate entity, and there is concomitant use of several drugs (with or without adulterants and potentiating factors) at the same time. It is also important to note that not all people exposed to different nephrotoxins will develop kidney disease.

However, with the ever- growing global burden of use of recreational drugs and their dependence, it is of paramount importance that clinicians and investigators are aware of the harmful renal effects, as timely intervention and community education can slow the burden of the disease. Furthermore, further research into the pathophysiological mechanisms associated with these drugs will provide a deeper understanding of the renal toxicity.

## References

[1] Beth H. Key Substance Use and Mental Health Indicators in the United States: Results from the 2019 National Survey on Drug Use and Health. Rockville, 2020.

[2] Mansoor K Kheetan M Shahnawaz S. Systematic review of nephrotoxicity of drugs of abuse, 2005–2016. BMC Nephrol. 2017;18:379. 29287591 10.1186/s12882-017-0794-0 PMC5747941

[3] Kariisa M Scholl L Wilson N. Drug overdose deaths involving cocaine and psychostimulants with abuse potential – United States, 2003–2017. MMWR Morb Mortal Wkly Rep. 2019;68:388–95. 31048676 10.15585/mmwr.mm6817a3 PMC6541315

[4] Fine DM Garg N Haas M. Cocaine use and hypertensive renal changes in HIV-infected individuals. Clin J Am Soc Nephrol. 2007;2:1125–30. 17942770 10.2215/CJN.02450607

[5] Mochizuki Y Zhang M Golestaneh L. Acute aortic thrombosis and renal infarction in acute cocaine intoxication: a case report and review of literature. Clin Nephrol. 2003;60:130–3. 12940616 10.5414/cnp60130

[6] Fabbian F Pala M de Giorgi A. Left kidney: an unusual site of cocaine-related renal infarction: a case report. Eur Rev Med Pharmacol Sci. 2012;16(Suppl 1):30–3. 22582481

[7] Fogo A Superdock KR Atkinson JB. Severe arteriosclerosis in the kidney of a cocaine addict. Am J Kidney Dis. 1992;20:513–5. 1442765 10.1016/s0272-6386(12)70267-6

[8] Madhrira MM Mohan S Markowitz GS. Acute bilateral renal infarction secondary to cocaine-induced vasospasm. Kidney Int. 2009;76:576–80. 18946498 10.1038/ki.2008.550

[9] Bemanian S Motallebi M Nosrati SM. Cocaine-induced renal infarction: report of a case and review of the literature. BMC Nephrol. 2005;6:10. 16176587 10.1186/1471-2369-6-10 PMC1253515

[10] Edmondson DA Towne JB Foley DW. Cocaine-induced renal artery dissection and thrombosis leading to renal infarction. WMJ. 2004;103:66–9. 15696837

[11] Herzlich BC Arsura EL Pagala M. Rhabdomyolysis related to cocaine abuse. Ann Intern Med. 1988;109:335–6. 3395041 10.7326/0003-4819-109-4-335

[12] Vakde T Diaz M Uday K. Rapidly reversible multiorgan failure after ingestion of “Molly” (pure 3,4-methylenedioxymethamphetamine): a case report. J Med Case Rep. 2014;8:204. 24942782 10.1186/1752-1947-8-204 PMC4082171

[13] Beaumier M Rioult J-P Georges M. Mushroom poisoning presenting with acute kidney injury and elevated transaminases. Kidney Int Rep. 2019;4:877–81. 31194187 10.1016/j.ekir.2019.02.016 PMC6551513

[14] Veronese FV Dode RSO Friderichs M. Cocaine/levamisole-induced systemic vasculitis with retiform purpura and pauci-immune glomerulonephritis. Braz J Med Biol Res. 2016;49:e5244. 27119429 10.1590/1414-431X20165244 PMC4849970

[15] Carrara C Emili S Lin M. Necrotizing and crescentic glomerulonephritis with membranous nephropathy in a patient exposed to levamisole-adulterated cocaine. Clin Kidney J. 2016;9:234–8. 26985374 10.1093/ckj/sfv141 PMC4792616

[16] Garg L Gupta S Swami A. Levamisole/cocaine induced systemic vasculitis and immune complex glomerulonephritis. Case Rep Nephrol. 2015;2015:1–5. 10.1155/2015/372413 PMC4531184 26290761

[17] van der Veer T Pennings E Tervaert JWC. Levamisole-contaminated cocaine: a hairy affair: figure 1. BMJ Case Rep. 2015;2015:bcr2015210970. 10.1136/bcr-2015-210970 PMC4550939 26311010

[18] Gu X Herrera GA. Thrombotic microangiopathy in cocaine abuse-associated malignant hypertension: report of 2 cases with review of the literature. Arch Pathol Lab Med. 2007;131:1817–20. 18081441 10.5858/2007-131-1817-TMICAM

[19] Botros M Salloum IM. Acute kidney injury associated with alcohol and cocaine abuse: a case report. Addict Disord Their Treat. 2016;15:49–51.

[20] Doctora JS Williams CW Bennett CR. Rhabdomyolysis in the acutely cocaine- intoxicated patient sustaining maxillofacial trauma: report of a case and review of the literature. J Oral Maxillofac Surg. 2003;61:964–7. 12905452 10.1016/s0278-2391(03)00241-6

[21] Roth D Alarcón FJ Fernandez JA. Acute rhabdomyolysis associated with cocaine intoxication. N Engl J Med. 1988;319:673–7. 3412385 10.1056/NEJM198809153191103

[22] Terada Y Shinohara S Matui N. Amphetamine-indeced myoglobinuric acute renal failure. Jpn J Med. 1988;27:305–8. 3057269 10.2169/internalmedicine1962.27.305

[23] Fahal IH Sallomi DF Yaqoob M. Acute renal failure after ecstasy. BMJ. 1992;305:29. 10.1136/bmj.305.6844.29 PMC1882520 1353389

[24] Ginsberg MD Hertzman M Schmidt-Nowara WW. Amphetamine intoxication with coagulopathy, hyperthermia, and reversible renal failure. Ann Intern Med. 1970;73:81–5. 5433281 10.7326/0003-4819-73-1-81

[25] Kendrick WC Hull AR Knochel JP. Rhabdomyolysis and shock after intravenous amphetamine administration. Ann Intern Med. 1977;86:381–7. 848798 10.7326/0003-4819-86-4-381

[26] Foley RJ Kapatkin K Verani R. Amphetamine-induced acute renal failure. South Med J. 1984;77:258–60. 6701599 10.1097/00007611-198402000-00035

[27] Bingham C. Necrotizing renal vasculopathy resulting in chronic renal failure after ingestion of methamphetamine and 3,4-methylenedioxymethamphetamine (“ecstasy”). Nephrol Dial Transplant. 1998;13:2654–5. 9794581 10.1093/ndt/13.10.2654

[28] Eldehni MT Roberts ISD Naik R. Case report of ecstasy-induced renal venous thrombosis. Clin Kidney J. 2010;3:459–60. 10.1093/ndtplus/sfq088 PMC4421714 25984053

[29] Cherney DZI Davids MR Halperin ML. Acute hyponatraemia and “ecstasy”: insights from a quantitative and integrative analysis. QJM. 2002;95:475–83. 12096153 10.1093/qjmed/95.7.475

[30] Traub SJ Hoffman RS Nelson LS. The “Ecstasy” hangover: hyponatremia due to 3,4-methylenedioxymethamphetamine. J Urban Health. 2002;79:549–55. 12468674 10.1093/jurban/79.4.549 PMC3456731

[31] Hartung TK Schofield E Short AI. Hyponatraemic states following 3,4-methylenedioxymethamphetamine (MDMA, ’ecstasy’) ingestion. QJM. 2002;95:431–7. 12096147 10.1093/qjmed/95.7.431

[32] Kwon C Zaritsky A Dharnidharka VR. Transient proximal tubular renal injury following Ecstasy ingestion. Pediatr Nephrol. 2003;18:820–2. 12774221 10.1007/s00467-003-1164-7

[33] Hallock RM Dean A Knecht ZA. A survey of hallucinogenic mushroom use, factors related to usage, and perceptions of use among college students. Drug Alcohol Depend. 2013;130:245–8. 23265089 10.1016/j.drugalcdep.2012.11.010

[34] Manner I Sagedal S Røger M. Renal amyloidosis in intravenous heroin addicts with nephrotic syndrome and renal failure. Clin Nephrol. 2009;72:224–8. 19761730 10.5414/cnp72224

[35] Raff E Halloran PF Kjellstrand CM. Renal failure after eating “magic” mushrooms. CMAJ. 1992;147:1339–41. 1302482 PMC1336442

[36] Bickel M Ditting T Watz H. Severe rhabdomyolysis, acute renal failure and posterior encephalopathy after magic mushroom abuse. Eur J Emerg Med. 2005;12:306–8. 16276262 10.1097/00063110-200512000-00011

[37] Kilcoyne MM Daly JJ Gocke DJ. Nephrotic syndrome in heroin addicts. Lancet. 1972;299:17–20. 10.1016/s0140-6736(72)90007-4 4108817

[38] Patel R Das M Palazzolo M. Myoglobinuric acute renal failure in phencyclidine overdose: report of observations in eight cases. Ann Emerg Med. 1980;9:549–53. 7436062 10.1016/s0196-0644(80)80222-8

[39] Akmal M Valdin JR McCarron MM. Rhabdomyolysis with and without acute renal failure in patients with phencyclidine intoxication. Am J Nephrol. 1981;1:91–6. 7349047 10.1159/000166498

[40] Uzan M Volochine L Rondeau E. [Renal disease associated with heroin abuse]. Nephrologie. 1988;9:217–21. 3216943

[41] Selby NM Anderson J Bungay P. Obstructive nephropathy and kidney injury associated with ketamine abuse. Clin Kidney J. 2008;1:310–2. 10.1093/ndtplus/sfn054 PMC4421282 25983920

[42] Chen C-H Lee M-H Chen Y-C. Ketamine-snorting associated cystitis. J Formos Med Assoc. 2011;110:787–91. 22248834 10.1016/j.jfma.2011.11.010

[43] Lai Y Wu S Ni L. Ketamine-associated urinary tract dysfunction: an underrecognized clinical entity. Urol Int. 2012;89:93–6. 22710265 10.1159/000338098

[44] Shahani R Streutker C Dickson B. Ketamine-associated ulcerative cystitis: a new clinical entity. Urology. 2007;69:810–2. 17482909 10.1016/j.urology.2007.01.038

[45] Harrington P Ali G Chan A. The development of focal segmental glomerulosclerosis secondary to anabolic steroid abuse. Case Rep. 2011;2011:bcr0720114531. 10.1136/bcr.07.2011.4531 PMC3233923 22669525

[46] Lee H-Y Hsu Y-C Hsu C-Y. Upper urinary tract damage caused by ketamine snorting—a report of nine cases. Urol Sci. 2015;26:182–5.

[47] Chu PSK Kwok SC Lam KM. ’Street ketamine’-associated bladder dysfunction: a report of ten cases. Hong Kong Med J. 2007;13:311–3. 17592176

[48] Thakur V Godley C Weed S. Cocaine-associated accelerated hypertension and renal failure. Am J Med Sci. 1996;312:295–8. 8969619 10.1097/00000441-199612000-00008

[49] Almukhtar SE Abbas AA Muhealdeen DN. Acute kidney injury associated with androgenic steroids and nutritional supplements in bodybuilders. Clin Kidney J. 2015;8:415–9. 26251708 10.1093/ckj/sfv032 PMC4515889

[50] Sandhu RS Como JJ Scalea TS. Renal failure and exercise-induced rhabdomyolysis in patients taking performance-enhancing compounds. J Trauma. 2002;53:761–4. 12394880 10.1097/00005373-200210000-00024

[51] Habscheid W Abele U Dahm HH. Schwere Cholestase mit Nierenversagen durch Anabolika bei einem Bodybuilder. . DMW Dtsch Med Wochenschr. 2008;124:1029–32. 10.1055/s-2007-1024477 10506840

[52] Daher EF Silva Júnior GB Queiroz AL. Acute kidney injury due to anabolic steroid and vitamin supplement abuse: report of two cases and a literature review. Int Urol Nephrol. 2009;41:717–23. 19387860 10.1007/s11255-009-9571-8

[53] Luciano RL Castano E Moeckel G. Bile acid nephropathy in a bodybuilder abusing an anabolic androgenic steroid. Am J Kidney Dis. 2014;64:473–6. 24953892 10.1053/j.ajkd.2014.05.010

[54] Alkhunaizi AM ElTigani MA Rabah RS. Acute bile nephropathy secondary to anabolic steroids. Clin Nephrol. 2016;85:121–6. 26587777 10.5414/CN108696

[55] Rao TKS Nicastri AD Friedman EA. Natural history of heroin-associated nephropathy. N Engl J Med. 1974;290:19–23. 4128109 10.1056/NEJM197401032900105

[56] Sahni V Garg D Garg S. Unusual complications of heroin abuse: transverse myelitis, rhabdomyolysis, compartment syndrome, and ARF. Clin Toxicol. 2008;46:153–5. 10.1080/15563650701639071 17917867

[57] do Sameiro Faria M. Nephropathy associated with heroin abuse in Caucasian patients. Nephrol Dial Transplant. 2003;18:2308–13. 14551358 10.1093/ndt/gfg369

[58] Turgutalp K Kiykim A Karabulut U. Reversible minimal change nephrotic syndrome and glomerular IgA deposition associated with nonparenteral heroin abuse: a case report. Med Princ Pract. 2012;21:492–4. 22539034 10.1159/000337941

[59] Tan AU Cohen AH Levine BS. Renal amyloidosis in a drug abuser. J Am Soc Nephrol. 1995;5:1653–8. 7780053 10.1681/ASN.V591653

[60] Bautista JEK Merhi B Gregory O. Heroin crystal nephropathy. Clin Kidney J. 2015;8:339–42. 26034599 10.1093/ckj/sfv018 PMC4440465

[61] May DC Helderman JH Eigenbrodt EH. Chronic Sclerosing Glomerulopathy (Heroin-Associated Nephropathy) in intravenous T’s and blues abusers. Am J Kidney Dis. 1986;8:404–9. 3812469 10.1016/s0272-6386(86)80166-4

[62] Yu L Jabr FI. Thrombotic microangiopathy associated with Opana Er intravenous abuse: a case report. Lebanese Med J. 2016;64:40–2. 10.12816/0023831 27169165

[63] Miller PJ Farland AM Knovich MA. Successful treatment of intravenously abused oral Opana ER-induced thrombotic microangiopathy without plasma exchange. Am J Hematol. 2014;89:695–7. 24668845 10.1002/ajh.23720

[64] Taverner D Harrison DJ Bell GM. Acute renal failure due to interstitial nephritis induced by “Glue-Sniffing” with subsequent recovery. Scott Med J. 1988;33:246–7. 3399879 10.1177/003693308803300208

[65] Moss AH. Fanconi’s syndrome and distal renal tubular acidosis after glue sniffing. Ann Intern Med. 1980;92:69. 7350875 10.7326/0003-4819-92-1-69

[66] Patel R Benjamin J. Renal disease associated with toluene inhalation. J Toxicol Clin Toxicol. 1986;24:213–23. 3014162 10.3109/15563658608990459

[67] Cámara-Lemarroy CR Gónzalez-Moreno EI Rodriguez-Gutierrez R. Clinical presentation and management in acute toluene intoxication: a case series. Inhal Toxicol. 2012;24:434–8. 22642292 10.3109/08958378.2012.684364

[68] Dasgupta A. Abuse of Magic Mushroom, Peyote Cactus, LSD, Khat, and Volatiles. Critical Issues in Alcohol and Drugs of Abuse Testing, Elsevier 2019, p. 477–94.

[69] Regunath H Ariyamuthu VK Dalal P. Bath salt intoxication causing acute kidney injury requiring hemodialysis. Hemodial Int. 2012;16:S47–9. 23036036 10.1111/j.1542-4758.2012.00750.x

[70] McNeely J Parikh S Valentine C. Bath salts: a newly recognized cause of acute kidney injury. Case Rep Nephrol. 2012;2012:1–5. 10.1155/2012/560854 PMC3914251 24555135

[71] Sutamtewagul G Sood V Nugent K. Sympathomimetic syndrome, choreoathetosis, and acute kidney injury ******following “bath salts” injection. Clin Nephrol. 2014;81:63–6. 24356039 10.5414/cn107559

[72] Rhidian R Babu A. Acute kidney injury requiring haemodialysis following ingestion of mephedrone. Case Rep. 2013;2013:bcr2012007974. 10.1136/bcr-2012-007974 PMC3618762 23456157

[73] Adebamiro A Perazella MA. Recurrent acute kidney injury following bath salts intoxication. Am J Kidney Dis. 2012;59:273–5. 22119408 10.1053/j.ajkd.2011.10.012

[74] Borek HA Holstege CP. Hyperthermia and multiorgan failure after abuse of “Bath Salts” containing 3,4-methylenedioxypyrovalerone. Ann Emerg Med. 2012;60:103–5. 22387085 10.1016/j.annemergmed.2012.01.005

[75] Ambruzs JM Serrell PB Rahim N. Thrombotic microangiopathy and acute kidney injury associated with intravenous abuse of an oral extended-release formulation of oxymorphone hydrochloride: kidney biopsy findings and report of 3 cases. Am J Kidney Dis. 2014;63:1022–6. 24529995 10.1053/j.ajkd.2014.01.015

[76] Cunningham EE Venuto RC Zielezny MA. Adulterants in heroin/cocaine: Implications concerning heroin-associated nephropathy. Drug Alcohol Depend. 1984;14:19–22. 6435983 10.1016/0376-8716(84)90014-0

[77] Kazory A Aiyer R. Synthetic marijuana and acute kidney injury: an unforeseen association. Clin Kidney J. 2013;6:330–3. 26064495 10.1093/ckj/sft047 PMC4400490

[78] Curtis B Mahat B Macklin M. Acute kidney injury related to intoxication from synthetic cannabis: don’t you know that you’re toxic? Cureus. 2022;14:e23427. 35481311 10.7759/cureus.23427 PMC9033635

[79] Zhao A Tan M Maung A. Rhabdomyolysis and acute kidney injury requiring dialysis as a result of concomitant use of atypical neuroleptics and synthetic cannabinoids. Case Rep Nephrol. 2015;2015:1–4. 10.1155/2015/235982 PMC4621326 26550500

[80] Bhanushali GK Jain G Fatima H. AKI associated with synthetic cannabinoids: a case series. Clin J Am Soc Nephrol. 2013;8:523–6. 23243266 10.2215/CJN.05690612 PMC3613952

[81] Mahmoud Kamel BT. A case of acute kidney injury and calcium oxalate deposition associated with synthetic cannabinoids. Saudia J Kidney Dis Transpl. 2015;26:802–3. 10.4103/1319-2442.160222 26178563

[82] Buser GL Gerona RR Horowitz BZ. Acute kidney injury associated with smoking synthetic cannabinoid. Clin Toxicol. 2014;52:664–73. 10.3109/15563650.2014.932365 25089722

[83] Gudsoorkar VS Perez Jr JA A new differential diagnosis: synthetic cannabinoids-associated acute renal failure. Methodist Debakey Cardiovasc J 2015;11:189. 26634029 10.14797/mdcj-11-3-189 PMC4666428

[84] Sherpa D Paudel BM Subedi BH. Synthetic cannabinoids: the multi-organ failure and metabolic derangements associated with getting high. J Community Hosp Intern Med Perspect. 2015;5:27540. 26333853 10.3402/jchimp.v5.27540 PMC4558292

[85] Thornton SL Wood C Friesen MW. Synthetic cannabinoid use associated with acute kidney injury*. Clin Toxicol. 2013;51:189–90. 10.3109/15563650.2013.770870 23473465

[86] Gupta A Kuperman M Shah S. N-methylamphetamine (“crystal meth”)−associated acute renal cortical necrosis. Kidney Int Rep. 2018;3:1473–6. 30450474 10.1016/j.ekir.2018.07.003 PMC6224663

[87] di Paolo N Fineschi V di Paolo M. Kidney vascular damage and cocaine. Clin Nephrol. 1997;47:298–303. 9181276

[88] Jaffe JA Kimmel PL. Chronic nephropathies of cocaine and heroin abuse: a critical review. Clin J Am Soc Nephrol. 2006;1:655–67. 17699270 10.2215/CJN.00300106

[89] Dunea G Arruda JAL Bakir AA. Role of cocaine in end-stage renal disease in some hypertensive African Americans. Am J Nephrol. 1995;15:5–9. 7872365 10.1159/000168794

[90] Mitra SC Seshan SV Salcedo JR. Maternal cocaine abuse and fetal renal arteries: a morphometric study. Pediatr Nephrol. 2000;14:315–8. 10775076 10.1007/s004670050766

[91] Mitra SC. Effect of cocaine on fetal kidney and bladder function. J Matern Fetal Med. 1999;8:262–9. 10582860 10.1002/(SICI)1520-6661(199911/12)8:6<262::AID-MFM6>3.0.CO;2-C

[92] Larocque A Hoffman RS. Levamisole in cocaine: unexpected news from an old acquaintance. Clin Toxicol. 2012;50:231–41. 10.3109/15563650.2012.665455 22455354

[93] Schürer S Klingel K Sandri M. Clinical characteristics, histopathological features, and clinical outcome of methamphetamine-associated cardiomyopathy. JACC Heart Fail. 2017;5:435–45. 28571597 10.1016/j.jchf.2017.02.017

[94] Peces R Navascués RA Baltar J. Antiglomerular basement membrane antibody-mediated glomerulonephritis after intranasal cocaine use. Nephron. 1999;81:434–8. 10095180 10.1159/000045328

[95] Mokhtari T Sheikhazadi A Hassanzadeh G. Potential adverse effects of amphetamines on kidney; a narrative review on current knowledge. J Renal Inj Prev. 2018;7:218–23.

[96] Pendergraft WF Herlitz LC Thornley-Brown D. Nephrotoxic effects of common and emerging drugs of abuse. Clin J Am Soc Nephrol. 2014;9:1996–2005. 25035273 10.2215/CJN.00360114 PMC4220747

[97] Key substance use and mental health indicators in the United States: Results from the 2018 National Survey on Drug Use and Health (HHS Publication No. PEP19-5068, NSDUH Series H-54). 2019.

[98] Berrens Z Lammers J White C. Rhabdomyolysis after LSD ingestion. Psychosomatics. 2010;51:356–356.e3. 20587768 10.1176/appi.psy.51.4.356

[99] Bora F Yilmaz F Bora T. Ecstasy (MDMA) and its effects on kidneys and their treatment: a review. Iran J Basic Med Sci. 2016;19:1151–8. 27917269 PMC5126214

[100] Bey T Patel A. Phencyclidine intoxication and adverse effects: a clinical and pharmacological review of an illicit drug. Cal J Emerg Med. 2007;8:9–14. 20440387 PMC2859735

[101] Herlitz LC Markowitz GS Farris AB. Development of focal segmental glomerulosclerosis after anabolic steroid abuse. J Am Soc Nephrol. 2010;21:163–72. 19917783 10.1681/ASN.2009040450 PMC2799287

[102] Anawalt BD. Diagnosis and management of anabolic androgenic steroid use. J Clin Endocrinol Metab. 2019;104:2490–500. 30753550 10.1210/jc.2018-01882 PMC6517163

[103] Mallappallil M Sabu J Friedman E. What do we know about opioids and the kidney? Int J Mol Sci. 2017;18:223. 28117754 10.3390/ijms18010223 PMC5297852

[104] Cooper C Bilbao JE Said S. Serum amyloid A renal amyloidosis in a chronic subcutaneous (“skin popping”) heroin user. J Nephropathol. 2013;2:196–200. 24475449 10.12860/JNP.2013.31 PMC3891138

[105] Baskerville JR. Toluene induced hypokalaemia: case report and literature review. Emerg Med J. 2001;18:514–6. 11696523 10.1136/emj.18.6.514 PMC1725726

[106] Dargan PI Sedefov R Gallegos A. The pharmacology and toxicology of the synthetic cathinone mephedrone (4-methylmethcathinone). Drug Test Anal. 2011;3:454–63. 21755604 10.1002/dta.312

[107] Habboushe J Sedor J. Cannabinoid hyperemesis acute renal failure: a common sequela of cannabinoid hyperemesis syndrome. Am J Emerg Med. 2014;32:690.e1–2. 10.1016/j.ajem.2013.12.013 24418446

[108] Chua JT Argueta DA DiPatrizio NV. Endocannabinoid system and the kidneys: from renal physiology to injury and disease. Cannabis Cannabinoid Res. 2019;4:10–20. 31346545 10.1089/can.2018.0060 PMC6653784

[109] Centers for Disease Control and Prevention (CDC). Acute kidney injury associated with synthetic cannabinoid use – multiple states, 2012. MMWR Morb Mortal Wkly Rep. 2013;62:93–8. 23407124 PMC4604808

